# SteC is a *Salmonella* kinase required for SPI-2-dependent F-actin remodelling

**DOI:** 10.1111/j.1462-5822.2007.01010.x

**Published:** 2008-01

**Authors:** John Poh, Charlotte Odendall, Ad Spanos, Cliona Boyle, Mei Liu, Paul Freemont, David W Holden

**Affiliations:** 1 Department of Molecular Microbiology and Infection Flowers Building, London SW7 2AZ, UK; 2 Stem Cell Biology and Developmental Genetics, National Institute for Medical Research London NW7 1AA, UK; 3 Division of Molecular Biosciences Imperial College London, London SW7 2AZ, UK

## Abstract

*Salmonella enterica* serovar Typhimurium (*S.* Typhimurium) replicates inside mammalian cells within membrane-bound compartments called *Salmonella*-containing vacuoles. Intracellular replication is dependent on the activities of several effector proteins translocated across the vacuolar membrane by the *Salmonella* pathogenicity island 2 (SPI-2)-type III secretion system (T3SS). This is accompanied by the formation in the vicinity of bacterial vacuoles of an F-actin meshwork, thought to be involved in maintaining the integrity of vacuolar membranes. In this study, we investigated the function of the SPI-2 T3SS effector SteC. An *steC* mutant strain was not defective for intracellular replication or attenuated for virulence in mice. However, the *steC* mutant was defective for SPI-2-dependent F-actin meshwork formation in host cells, although the vacuolar membranes surrounding mutant bacteria appeared to be normal. Expression of SteC in fibroblast cells following transfection caused extensive rearrangements of the F-actin cytoskeleton. Sequence analysis identified amino acid similarity between SteC and the human kinase Raf-1. A His-tagged SteC fusion protein had kinase activity *in vitro* and a point mutant lacking kinase activity was unable to induce F-actin rearrangements *in vivo*. We conclude that SPI-2-dependent F-actin meshwork formation depends on the kinase activity of SteC, which resembles more closely eukaryotic than prokaryotic kinases.

## Introduction

Following uptake by host cells, *Salmonella enterica* serovar Typhimurium (*S.* Typhimurium) replicates within a membrane-bound compartment, the *Salmonella*-containing vacuole (SCV). Numerous bacterial genes are required for intracellular survival, replication and virulence of this pathogen in mice. These include a multifunctional virulence system called the *Salmonella* pathogenicity island-2 (SPI-2) type III secretion system (T3SS; Waterman and Holden, 2003). The SPI-2 T3SS is induced intracellularly (Cirillo *et al*., 1998) and translocates several effectors into the vacuolar membrane and host cell cytosol (Waterman and Holden, 2003). These effectors are involved in several physiological activities, including the regulation of vacuolar membrane dynamics (Ruiz-Albert *et al*., 2002; Boucrot *et al*., 2005; Henry *et al*., 2006), inducing motility of infected cells (Worley *et al*., 2006), targeting SCVs to the Golgi apparatus in epithelial cells (Salcedo and Holden, 2003), and formation of an actin cytoskeleton meshwork around SCVs (Méresse *et al*., 2001; Unsworth *et al*., 2004).

At least two effectors, SseF and SseG, are encoded within SPI-2, but several others are encoded by genes located at different sites in the bacterial chromosome, and the full repertoire of effectors is unknown (Waterman and Holden, 2003; Kujat Choy *et al*., 2004; Geddes *et al*., 2005). The expression of SPI-2 genes and genes encoding effectors located outside the pathogenicity island requires the SPI-2-encoded two-component regulatory system SsrA–SsrB (Cirillo *et al*., 1998). In recent work we used a DNA microarray of *S.* Typhimurium to compare the levels of mRNAs in wild-type and *ssrA* mutant bacteria grown in conditions that result in strong expression of the SsrA–SsrB regulon. This led to the identification of an effector (SseL) with deubiquitinase activity (Rytkönen *et al*., 2007). Another gene whose RNA level was significantly lower in the *ssrA* mutant compared with the wild-type strain is *STM1698* (Rytkönen *et al*., 2007)*. STM1698* was previously identified in a signature-tagged mutagenesis screen as a gene important in colonization of the chick intestine (Morgan *et al*., 2004). A subsequent study by Geddes *et al*. (2005) showed that the product of *STM1698* is translocated into host cells in a SPI-2 T3SS-dependent manner, and the gene was designated *steC* (*Salmonella* translocated effector C). In this study we have further characterized the product of *steC*. We show that SteC is a kinase that is required for SPI-2 T3SS-dependent actin meshwork formation in infected cells.

## Results and discussion

### SsrA-dependent intracellular expression of *steC*

**Fig. 1:**
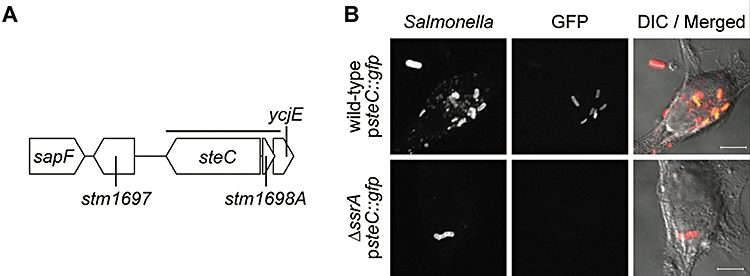
A. Map of the chromosomal region encompassing *steC* in *S.* Typhimurium. Black bar indicates the 1678 bp region containing the *steC* promoter and open reading frame used for constructing the *gfp* fusion. B. Intracellular expression of *steC::gfp* is dependent on *ssrA*. Confocal immunofluorescence analysis of HeLa cells infected with wild-type or *ssrA* mutant bacteria carrying plasmid-borne *steC::gfp* fusions and fixed 8 h post invasion. Bacteria were detected with an anti-*Salmonella* antibody (red in merged image). The upper panel shows an extracellular bacterium not expressing GFP and several intracellular bacteria expressing GFP. Bars represent 5 μm.

A map of the chromosomal region encompassing *steC* is shown in Fig. 1A. To determine if intracellular expression of *steC* is regulated by SsrA–B, the *steC* open reading frame and 300 bp of DNA upstream from its start codon (see bar in Fig. 1A) was fused to a promoterless *gfp* gene. The fusion was ligated into a plasmid and introduced into wild-type or *ssrA* mutant strains, which were then used to infect HeLa cells. Infected cells were fixed at 2 h intervals following invasion, and examined by fluorescence and differential interference contrast (DIC) microscopy. Reporter activity was detected in intracellular but not extracellular wild-type bacteria, 8 h post invasion (Fig. 1B). No expression was detected in the *ssrA* mutant strain, confirming that *steC* is part of the SsrA–B regulon.

### Secretion and translocation of SteC-2HA

**Fig. 2:**
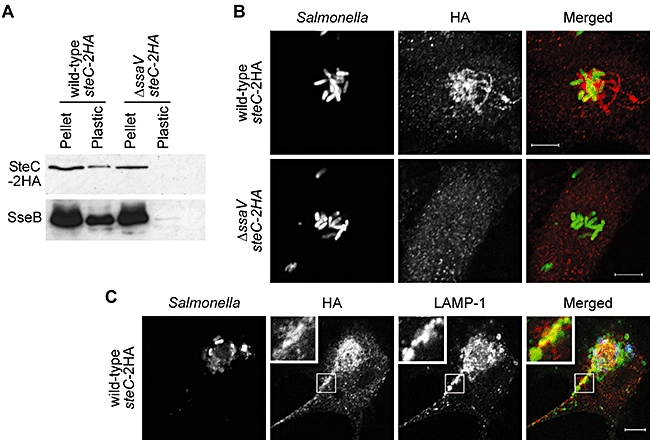
Intracellular translocation of SteC-2HA requires a functional SPI-2 T3SS. A. *In vitro* secretion of SteC-2HA. Wild-type and *ssaV* mutant strains expressing 2HA-tagged SteC were grown overnight in SPI-2-inducing conditions and bacterial cell pellet and extracellular proteins (plastic) were analysed by immunoblotting. B and C. Localization of translocated SteC-2HA. HeLa cells were infected for 8 h with *S.* Typhimurium strains expressing 2HA-tagged SteC from the chromosome. Cells were fixed and immunolabelled to detect (B) the HA-epitope tag (red in merged image) and *Salmonella* (green in merged image) or (C) the HA-epitope tag (red in merged image), *Salmonella* (blue in merged image) and LAMP-1 (green in merged image). Boxed insets in (C) are magnifications of indicated regions showing colocalization between SteC-2HA and LAMP-1 on Sifs. Bars represent 5 μm.

To detect secreted and translocated SteC, a gene encoding a double haemagglutinin (2HA) epitope-tagged version of SteC (*steC-2HA*) was introduced into the chromosome in place of the *steC* allele in the wild-type strain and an isogenic strain carrying a mutation in *ssaV*, which encodes an essential component of the SPI-2 T3SS (Beuzón *et al*., 1999). Wild-type and *ssaV* mutant strains containing *steC-2HA* were grown in magnesium minimal MES medium (MgM-MES) at pH 5.0, which induces the expression of the SPI-2 T3SS and secretion of its effectors (Beuzón *et al*., 1999). Under these conditions, SPI-2 T3SS-secreted proteins accumulate on the plastic surface of the tube in which the bacteria are grown (Beuzón *et al*., 1999; Yu *et al*., 2004). Proteins were recovered from this location and from the bacterial cell pellet, separated by SDS-PAGE and analysed by immunoblotting (Fig. 2A). Rabbit anti-SseB antibody (Beuzón *et al*., 1999) was used as a positive control. SteC-2HA was detected extracellularly when expressed in the wild-type strain background, but was found only in the bacterial cell pellet when expressed in the *ssaV* mutant strain background. This shows that *S.* Typhimurium requires a functional SPI-2 T3SS to secrete SteC *in vitro*.

To examine the translocation of SteC-2HA in host cells, HeLa cells were infected with wild-type or *ssaV* mutant strains containing *steC-2HA*. At 8 h post invasion, cells were fixed, permeabilized with saponin to allow detection of translocated protein (Yu *et al*., 2004) and immunolabelled with anti-*Salmonella* and anti-HA antibodies. SteC-2HA was readily detectable in HeLa cells infected with wild-type bacteria, whereas cells infected with the *ssaV* mutant did not show any immunolabelling with the anti-HA antibody (Fig. 2B). This indicates that SteC requires a functional SPI-2 T3SS to be translocated into host cells. Cells infected with wild-type bacteria expressing SteC-2HA were also labelled for LAMP-1, a lysosomal membrane glycoprotein abundant on the SCV membrane and *Salmonella*-induced filaments (Sifs), which are tubules that extend from SCVs in infected epithelial cells (Garcia-del Portillo *et al*., 1993). There was extensive colocalization between SteC-2HA and SCV- and Sif-associated LAMP-1 (Fig. 2C). Therefore, SteC is a translocated SPI-2 effector that shows a similar localization pattern to that of several other SPI-2 effectors (Waterman and Holden, 2003).

### Intracellular replication and virulence assays

The SPI-2 T3SS is required for replication of *S.* Typhimurium inside host cells (Waterman and Holden, 2003). Therefore, an *steC* knock-out mutant was constructed to investigate its intracellular growth, compared with that of the wild-type strain and an *ssaV* mutant. Replication assays were performed in epithelial (HeLa) cells and RAW macrophages. At 2 h and 16 h post uptake in each cell type, the growth of the *steC* mutant was indistinguishable from that of the wild-type strain, while the *ssaV* mutant displayed a strong replication defect in both cell types (data not shown). To determine the importance of SteC for virulence in the mouse model of systemic infection, the *steC* mutant strain was subjected to a virulence test involving mixed infections of mice. A competitive index (CI), which provides a value for the relative degree of virulence attenuation, was determined after recovering bacteria from spleens of infected animals, 48 h after intraperitoneal (i.p.) inoculation (Beuzón and Holden, 2001). The CI for the *steC* mutant strain versus the wild-type strain was 1.15%± 0.08%, consistent with results of a previous study (Geddes *et al*., 2005). Furthermore, we failed to detect a virulence defect of the *steC* mutant when the mixed inoculum was administered by the oral route (data not shown).

### SteC is required for SPI-2-dependent F-actin meshwork formation by intracellular bacteria

**Fig. 3:**
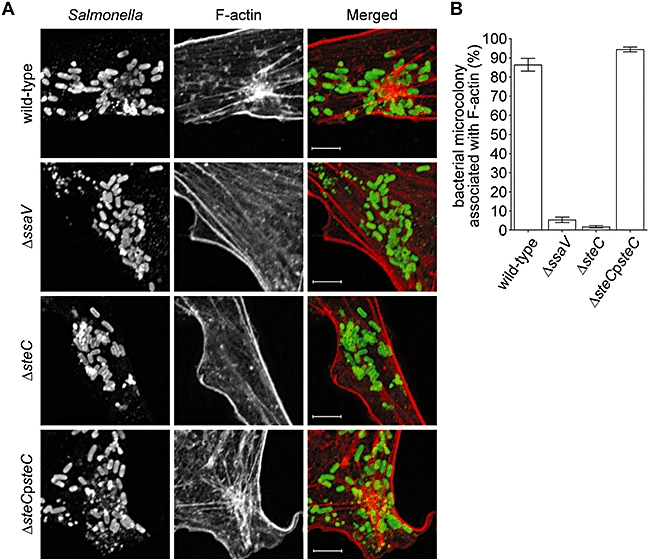
SteC is required for SPI-2-dependent F-actin reorganization. Swiss 3T3 cells were infected for 8 h with wild-type, *ssaV* mutant, *steC* mutant or *steC* mutant strains carrying a functional allele. A. Representative confocal images of cells immunolabelled to detect *Salmonella* (green in merged image). F-actin was visualized by phalloidin-RRX staining (red in merged image). Bars represent 5 μm. B. Quantification of F-actin remodelling by *Salmonella* strains. Results are the means ± SE of three independent experiments, in which a total of 300 infected cells were examined for each strain.

One characteristic of the SPI-2 T3SS, for which the corresponding effector(s) has not been identified, is the formation of an F-actin meshwork around SCVs (Méresse *et al*., 2001). Therefore, F-actin meshwork formation of the *steC* mutant was investigated. Swiss 3T3 fibroblasts, in which the SPI-2-dependent F-actin phenotype is particularly well defined (Méresse *et al*., 2001), were infected for 8 h with different strains, then fixed and labelled to detect *Salmonella* and F-actin. In wild-type-infected cells, 86.4% ± 3.2% of microcolonies were associated with a dense meshwork of F-actin, compared with 5.2% ± 1.3% in cells infected with the *ssaV* mutant (Fig. 3A and B). Deletion of *steC* reduced the numbers of microcolonies associated with F-actin to 1.7% ± 0.6%. However, if the *steC* mutant carried a functional *steC* allele under the control of its own promoter on a plasmid, 94.3% ± 1.2% of microcolonies displayed an F-actin meshwork (Fig. 3A and B). These results show that SteC is required for the formation of the SPI-2-dependent F-actin meshwork.

### Role of SteC in vacuole membrane integrity

Previous work from our laboratory has implicated SPI-2 T3SS-dependent F-actin reorganization in maintaining the integrity of the *Salmonella* vacuolar membrane (Méresse *et al*., 2001). We therefore investigated the integrity of intracellular vacuoles enclosing the *steC* mutant strain. RAW macrophages were infected for 12 h with GFP-expressing wild-type, *steC* or *sifA* mutant bacteria. The majority of *sifA* mutants lose their vacuolar membrane by 8 h post uptake (Beuzón *et al*., 2000) and therefore provide a positive control for vacuolar membrane loss. Cells were treated with digitonin to selectively permeabilize the host cell plasma membrane (Salcedo and Holden, 2003), and labelled with an anti-*Salmonella* antibody. Under these conditions, the majority (64.3%± 4.1%) of wild-type bacteria failed to label with the anti-*Salmonella* antibody, confirming the presence of an intact vacuolar membrane. However, only 28.6% ± 3.4% of the intracellular *sifA* mutant strain failed to label with the antibody, indicating that the majority had lost vacuolar membrane integrity. Labelling of the *steC* mutant by this method was similar to that of the wild-type strain (60.2% ± 3.3%). The integrity of vacuoles containing *steC* mutant bacteria was also confirmed using LAMP-1 labelling as a marker for the *Salmonella* vacuolar membrane (data not shown). Therefore, loss of SteC does not result in noticeable destabilization of the SCV.

The evidence indicating a role for SPI-2-associated F-actin in stability of the vacuolar membrane came from experiments in which membranes enclosing wild-type but not *ssaV* mutant bacteria were destabilized when infected cells were treated with the actin-depolymerizing drugs cytochalasin D or latrunculin B (Méresse *et al*., 2001). However, prolonged exposure of host cells to these drugs results in depolymerization of the vast majority of cellular F-actin. In view of the results described above, it would appear that the effects of these drugs on SCV membranes are indirect and unrelated to SteC-directed actin remodelling.

### SteC is a kinase

**Fig. 4:**
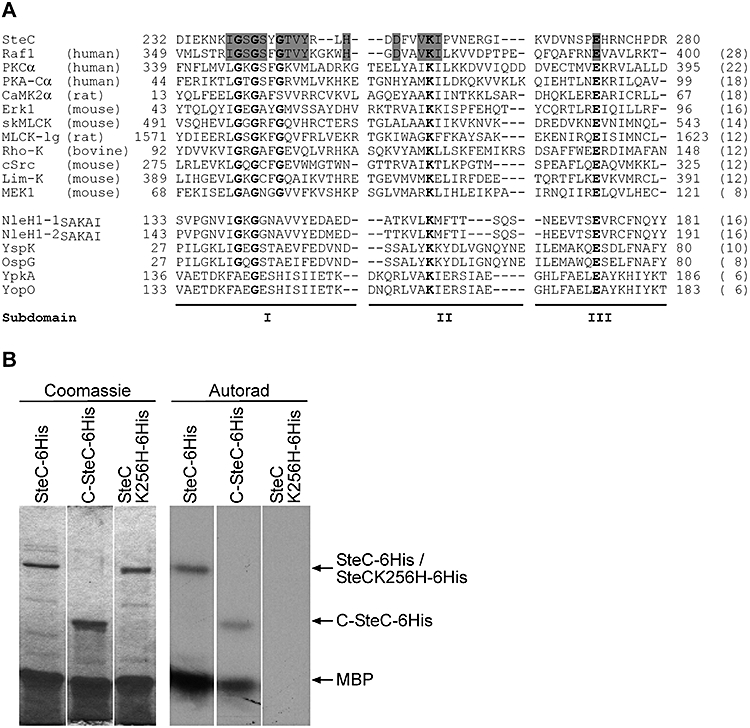
SteC is a kinase. A. Amino acid alignment of SteC with a selection of eukaryotic kinases (upper panel, adapted from Hanks and Hunter, 1995) and the following known or predicted bacterial kinases (lower panel): NleH1-1_SAKAI_ and NleH1-2_SAKAI_ from *E. coli* O157 (Tobe *et al*., 2006), YspK from *Yersinia enterocolitica* Biovar 1B (Matsumoto and Young, 2006), OspG from *Shigella flexneri* (Kim *et al*., 2005), YpkA from *Yersinia pseudotuberculosis* (Galyov *et al*., 1993) and YopO from *Yersinia enterocolitica* (Barz *et al*., 2000). The percentage identity between subdomains I–III of each kinase and SteC is shown in brackets on the far right. The highly conserved glycine-rich loop, lysine and glutamic acid residues found in the majority of kinases are indicated in bold. SteC is most similar to human Raf-1 and conserved residues between the two proteins are highlighted in grey. B. *In vitro* kinase assay. SteC, the lysine mutant (SteCK256H) and the kinase domain of SteC (C-SteC) were expressed as 6-His fusion proteins and incubated separately with the general kinase substrate MBP and [γ^−32^P]ATP. Proteins were subjected to SDS-PAGE followed by Coomassie blue staining and autoradiography.

*steC* is predicted to encode a protein of 457 amino acids, originally annotated as a putative inner membrane protein (McClelland *et al*., 2001). Functional predictions using the InterProScan search engine (http://www.ebi.ac.uk/InterProScan/) identified a region in SteC with similarity to kinases. Visual alignment of this region (aa 232–280) with several eukaryotic and prokaryotic kinases revealed residues in SteC (indicated in bold, Fig. 4A) that are highly conserved in subdomains I, II and III of kinases (Hanks *et al*., 1988). The consensus Gly-X-Gly-X-X-Gly is found in subdomain I of many kinases and functions as a nucleotide positioning motif that has a critical role in ATP binding (Bossemeyer, 1994). Subdomain II contains an invariant Lys residue that anchors ATP and contributes to the correct orientation of the triphosphate by interacting with the α- and β- phosphates (Hanks and Hunter, 1995). Mutation of this residue invariably results in a loss of kinase activity (Bossemeyer, 1993; Iyer *et al*., 2005). The nearly invariant Glu residue in subdomain III helps stabilize interactions between the Lys residue and ATP (Hanks and Hunter, 1995). The percentage identities of subdomains I-III of several kinases to SteC (Fig. 4A, far right brackets) indicate that this region of SteC has closest similarity to the human RAF proto-oncogene serine/threonine-protein kinase (Raf-1; Wellbrock *et al.*, 2004). However, SteC lacks the conserved central core of the catalytic domain in subdomains VI through IX of eukaryotic kinases, including highly conserved residues in the catalytic loop, and the His-Arg-Asp, Asp-Phe-Gly and Ala-Pro-Glu motifs, found in subdomains VI, VII and VIII respectively, which are important for phosphotransfer activity and substrate recognition of the kinase (Hanks *et al*., 1988; Hanks and Hunter, 1995).

To determine if SteC is a kinase, SteC, SteCK256H (in which the putative ATP-anchoring Lys residue in subdomain II was replaced by His) and C-SteC (lacking the N-terminal 200 amino acids, which show no similarity to kinases) were expressed and highly enriched as 6-His fusion proteins (Fig. 4B). These were incubated separately with [γ^−32^P]ATP and the general kinase substrate, myelin basic protein (MBP). Proteins were then separated by SDS-PAGE and analysed by autoradiography (Fig. 4B). SteC-6His and C-SteC-6His both phosphorylated MBP and also underwent autophosphorylation, a characteristic of many kinases (Hanks and Hunter, 1995; Morrison and Cutler, 1997; Chong *et al*., 2001). Mutation of Lys^256^ resulted in the complete loss of kinase activity. These results confirm that SteC is a kinase whose enzymatic activity is dependent on a conserved Lys residue in subdomain II, but not the N-terminal 200 amino acids of the protein. As for several other SPI-2 T3SS effectors, this region might be important for secretion, translocation and localization of SteC (Miao and Miller, 2000).

So far as we are aware, SteC is only the fourth T3SS effector to be identified displaying kinase activity. The others are OspG of *Shigella flexneri* (Kim *et al*., 2005), YopO/YpkA of the plasmid-encoded *Yersinia* T3SS (Galyov *et al*., 1993; Barz *et al*., 2000), and YspK, a recently identified effector of the chromosomally encoded Ysp T3SS of *Yersinia enterocolitica* (Matsumoto and Young, 2006). Of these kinases, OspG and YspK share 90% identity within subdomains I–III (data not shown). Two additional effectors, NleH1-1_SAKAI_ and NleH1-2_SAKAI_ from *Escherichia coli* O157, are also predicted to be kinases on the basis of strong similarity to OspG (Tobe *et al*., 2006). However, SteC and YopO/YpkA are not members of this subclass of bacterial kinases, and SteC has greater similarity to eukaryotic kinases, especially Raf-1.

### Intracellular F-actin meshwork formation by *Salmonella* is dependent on Lys^256^ of SteC

**Fig. 5:**
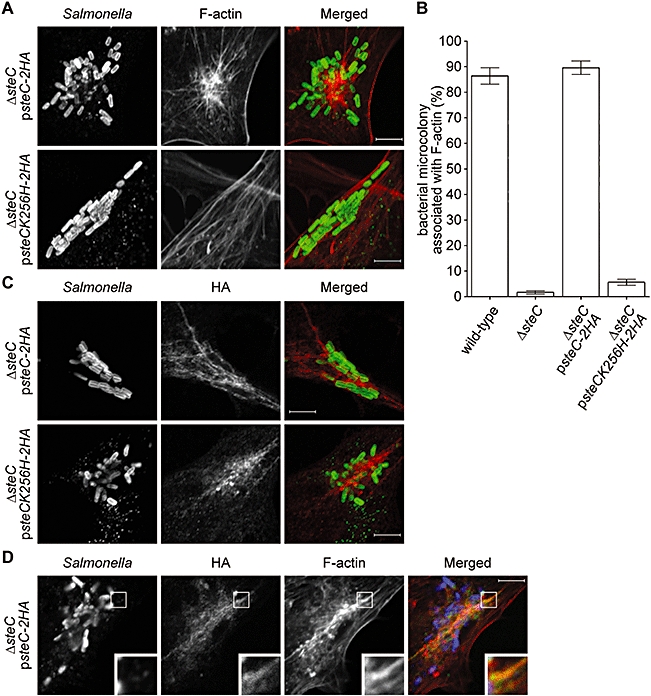
Intracellular F-actin reorganization by *Salmonella* is dependent on the Lys^256^ of SteC. A. Representative confocal images of Swiss 3T3 fibroblasts infected for 8 h with an *steC* mutant containing plasmid-borne *steC-2HA*, or *steCK256H-2HA*. Cells were immunolabelled to detect *Salmonella* (green in merged image). F-actin was visualized by phalloidin-RRX staining (red in merged image). Bars represent 5 μm. B. Quantification of F-actin reorganization by *Salmonella* strains. Results are the means ± SE of three independent experiments, in which a total of 300 infected cells were examined for each strain. Results for cells infected with wild-type or *steC* mutant strains are for comparison and are from Fig. 3B. C. Representative confocal images of infected cells showing translocation of SteC-2HA and SteCK256H-2HA expressed from a low-copy plasmid. Cells were fixed and immunolabelled to detect *Salmonella* (green in merged image) and HA (red in merged image). Bars represent 5 μm. D. Representative confocal micrograph of an infected cell showing translocated SteC-2HA localizing to F-actin. Cells were fixed and immunolabelled to detect *Salmonella* (blue in merged image) and HA (green in merged image. F-actin was visualized by phalloidin-RRX staining (red in merged image). Boxed insets are magnifications of indicated region showing localization of SteC-2HA with F-actin. Bars represent 5 μm.

To determine the importance of SteC Lys^256^ in F-actin meshwork formation by *Salmonella* in infected cells, Swiss 3T3 cells were infected with an *steC* mutant strain expressing one of two epitope-tagged versions of SteC (SteC-2HA or SteCK256H-2HA) from a plasmid. Infected cells were fixed at 8 h post invasion and labelled to detect *Salmonella* and F-actin (Fig. 5A), or *Salmonella* and the HA epitope (Fig. 5C). Mutation of Lys^256^ had no detectable effect on translocation of the protein (Fig. 5C). *Salmonella*-associated F-actin was detected in 89.5% ± 2.6% of cells infected with the strain expressing SteC-2HA, but only in 5.6% ± 1.2% of cells infected with the strain expressing SteCK256H-2HA (Fig. 5A and B). As Lys^256^ is essential for kinase activity of SteC, SPI-2 T3SS-dependent F-actin meshwork formation almost certainly requires the kinase activity of SteC. To determine the localization of SteC-2HA in infected Swiss 3T3 cells, cells were fixed at 8 h post invasion and labelled to detect *Salmonella*, HA and F-actin. A substantial degree of colocalization between SteC-2HA and F-actin was observed (Fig. 5D). This indicates that in addition to its localization on or close to the SCV membrane (Fig. 2C), SteC-2HA also localizes to SPI-2-induced F-actin structures.

### SteC is sufficient to cause ROCK-like F-actin reorganization

**Fig. 6:**
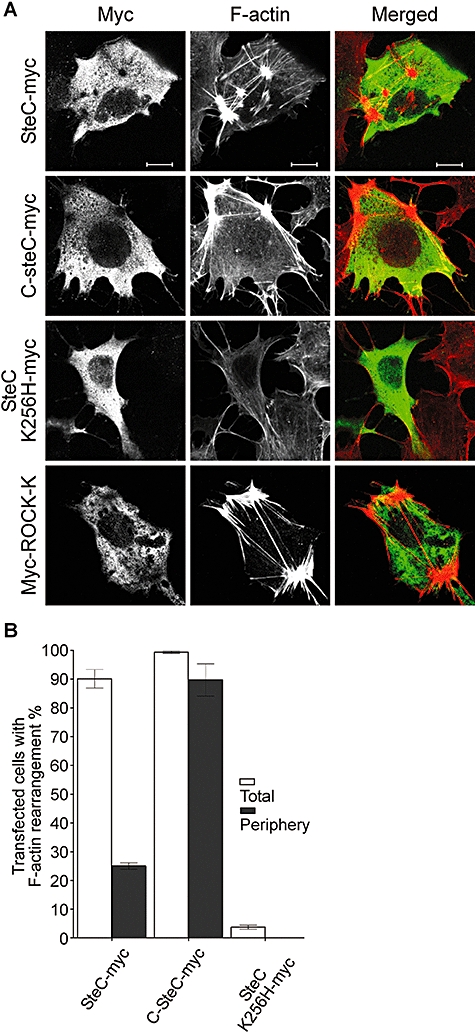
SteC is sufficient to cause ROCK-like F-actin reorganization. A. Representative confocal images of Swiss 3T3 cells transfected with vectors expressing myc-tagged SteC, C-SteC, SteCK256H or active ROCK (ROCK-K). Cells were immunolabelled with anti-Myc antibody (green in merged image) and F-actin was visualized by phalloidin-RRX staining (red in merged image). Bars represent 10 μm. B. Quantification of transfected cells displaying F-actin reorganization (white bars) and also periphery F-actin reorganization (black bars). Results are the means ± SE of three independent experiments, in which a total of 300 transfected cells were examined for each transfection vector.

We next investigated whether SteC activity is sufficient to cause F-actin reorganization in host cells. Swiss 3T3 fibroblasts were transfected with vectors encoding c-myc-epitope-tagged SteC (SteC-myc), the kinase-inactive point mutant (SteCK256H-myc) or the kinase domain (C-SteC-myc). Cells were transfected for 20 h in medium containing serum, and then incubated for another 3 h in serum-free medium before fixation, immunolabelling and examination by confocal microscopy. Untransfected fibroblasts or cells expressing SteCK256H-myc contained very few organized actin filaments except in the cortical region (Fig. 6A). In contrast, expression of SteC-myc or C-SteC-myc resulted in the formation of thick actin cables connecting large clusters of highly condensed F-actin (Fig. 6A and B). In cells expressing SteC-myc, these structures appeared to be randomly distributed throughout the cytoplasm, but in cells expressing C-SteC, clusters and cables were mainly found at the cell periphery (Fig. 6A and B). Therefore, SteC is sufficient to induce substantial reorganization of the host cell actin cytoskeleton in the absence of other bacterial effectors and its N-terminal 200 amino acids appear to have a role in its regulation or localization.

*Salmonella*-containing vacuole-associated F-actin has different morphologies, depending at least in part on the host cell type. These include a meshwork of F-actin between SCVs of a bacterial microcolony, a cage-like structure enclosing several SCVs (Méresse *et al*., 2001), or a highly condensed cluster of F-actin, frequently positioned towards the centre of a bacterial microcolony (Miao *et al*., 2003), from which long cables or filaments sometimes extend (Figs 3A and 5A). This phenotype is interesting in relation to the similarity displayed by SteC to Raf-1. Raf-1 plays a central role in cell proliferation, differentiation and survival. Raf kinases also influence actin cytoskeleton dynamics by modulating signalling pathways involving the Rho effector ROCK (Pritchard *et al*., 2004; Ehrenreiter *et al*., 2005; Castellani *et al*., 2006). The F-actin clusters and cables that are formed in fibroblasts following expression of SteC are very similar in appearance to those produced upon expression of active ROCK (Fig. 6A lower panel; Amano *et al*., 1997). However, treatment of infected cells with the ROCK inhibitor Y-27632 did not affect SteC-dependent F-actin remodelling around *Salmonella* microcolonies (data not shown). We are therefore currently investigating the possibility that SteC targets other component(s) of a pathway involving ROCK and other signalling pathways. Previous work from our laboratory has shown that several proteins involved in actin assembly, including Cdc42, Rac, N-WASP, Scar/WAVE and Arp2/3 are not involved in SPI-2 T3SS-dependent F-actin meshwork formation (Unsworth *et al*., 2004).

As SteC does not appear to contribute significantly to virulence in the murine model of systemic infection, the broader physiological significance of the kinase activity of SteC and its effect on the actin cytoskeleton is currently unknown. However, homologues of *steC* are present in other *S. enterica* serovars, including *S.* Paratyphi, *S.* Typhi and *S.* Choleraesuis (Geddes *et al*., 2005), and it is possible that SteC has a role in colonization of the chick intestine (Morgan *et al*., 2004) and/or other hosts.

## Experimental procedures

### Bacterial strains and growth conditions

Bacteria (Table 1) were grown in Luria–Bertani (LB) medium supplemented with carbenicillin (50 μg ml^−1^), kanamycin (50 μg ml^−1^) or chloramphenicol (30 μg ml^−1^), for strains resistant to these antibiotics (Amp^r^, Km^r^ and Cm^r^ respectively). To induce SPI-2 gene expression and SPI-2-dependent secretion, bacteria were grown in MgM-MES, containing 170 mM 2-[N-morpholinol]ethane-sulfonic acid (MES) at pH 5.0, 5 mM KCl, 7.5 mM (NH_4_)_2_SO_4_, 0.5 mM K_2_SO_4_, 1 mM KH_2_PO_4_, 8 μM MgCl_2_, 38 mM glycerol and 1% Casamino acids (Beuzón *et al*., 1999) with the corresponding antibiotics when appropriate. Bacteria were grown at 37°C overnight with aeration.

**Table 1:** Bacterial strains used in this study.

Strain	Description	Reference/source
12023	Wild-type *S*. Typhimurium	NTCC (Colindale, UK)
12023,p*steC*::gfp	*steC::gfp* under control of the *steC* promoter in 12023 (amp^r^)	This study
P3F4	*ssrA*::mTn*5* (km^r^)	Shea *et al*. (1996)
Rosetta	*E. coli* (cm^r^)	Novagen
Δ*ssrA*,p*steC*::gfp	*steC::gfp* under control of the *steC* promoter in P3F4 (km^r^, amp^r^)	This study
Δ*steC*	Δ*steC*::km in 12023 (km^r^)	This study
*steC*-2HA	*steC*-2HA in 12023 (km^r^)	This study
Δ*steC,*p*steC*	pWSK29*steC* in Δ*steC* (km^r^, amp^r^)	This study
Δ*steC,*p*steC*-2HA	pWSK29*steC*-2HA in Δ*steC* (km^r^, amp^r^)	This study
Δ*steC,*p*steCK256H-*2HA	pWSK29*steCK256H-*2HA in Δ*steC* (km^r^, amp^r^)	This study
HH119	*ssaV*::*aphT* (km^r^) in 12023	Deiwick *et al*. (1998)
Δ*ssaV,steC*-2HA	*steC*-2HA in HH119 (km^r^)	This study
TOP10	*E. coli*	Invitrogen

### Plasmids

Plasmids used in this study are listed in Table 2.

**Table 2:** Plasmids used in this study.

Plasmid	Description	Reference/source
pFPV25	Promoter trap vector, used to fuse promoters to the green fluorescent protein gene, *gfp*	Valdivia and Falkow (1996)
pFPV25.1	*rpsM::gfpmut3a* promoter fusion in pFPV25	Valdivia and Falkow (1996)
p*steC*::gfp	*steC* promoter and open reading frame fused to promoterless *gfp*	This study
pSU315	Template for HA-tagging of genes containing kanamycin cassette	Uzzau *et al*. (2001)
pWSK29	Low copy plasmid	Wang and Kushner (1991)
p*steC*	pWSK29 containing *steC*	This study
p*steC*-2HA	pWSK29 containing *steC*-2HA	This study
p*steCK256H-*2HA	pWSK29 containing *steCK256H-*2HA	This study
pRK5myc	Transfection vector containing multiple cloning site downstream of c-myc	Dr E Caron
pRK5*steCmyc*	*steC* tagged with N-terminal c-myc tag	This study
pRK5*steCK256Hmyc*	*steCK256H* tagged with N-terminal c-myc tag	This study
pRK5*C-steCmyc*	*C-steC* tagged with N-terminal c-myc tag	This study
Myc-ROCK-K	Active construct of ROCK tagged with N-terminal c-myc tag in pRK5myc	Dr E Caron Olazabal *et al*. (2002)
pET28b	Expression vector containing an N-terminal 6-His tag	Novagen
pET28b*steC-6His*	*steC* tagged with 6-His tag	This study
pET28b*steCK256H-6His*	*steCK256H* tagged with 6-His tag	This study
pET28b*C-steC-6His*	*C-steC* tagged with 6-His tag	This study

### Primers and construction of mutant strain, epitope tagging, site-directed mutagenesis and expression vectors

The primers used in this study are listed in Table S1. Polymerase chain reactions (PCRs) were performed using either the Taq polymerase (Sigma) or the Expand Long-Template PCR system (Roche) protocols. The Quikchange II site-directed mutagenesis kit protocol (Stratagene) was used to construct the Lys to His point mutant. The chromosomal deletion of *steC* in *S.* Typhimurium 12023 was performed by using the one-step gene-disruption technique (Datsenko and Wanner, 2000). The influenza virus HA epitope DNA sequence was fused to the chromosomal copy of *steC* according to Uzzau *et al*. (2001). *steC*-2HA was transduced by bacteriophage P22 into the *ssaV* mutant strain following the method of Davis *et al*. (1980). Site-directed mutagenesis was performed according to manufacturer's recommendations (Quikchange, Stratagene). Lys at residue 256 of SteC was changed to His using primers K256H-F and K256H-R. Primers for constructs are listed in Table S1.

### Antibodies and reagents

Anti-*Salmonella* goat polyclonal antibody CSA-1 (Kirkegaard and Perry Laboratories, Gaithersburg, MD) was used at a dilution of 1:200. Anti-HA mouse monoclonal antibody (HA.11; Covance) was used at a dilution of 1:200 (immunofluorescence microscopy) and 1:1000 (Western blot). Anti-HA rat monoclonal antibody (Roche) was used at a dilution of 1:200 for immunofluorescence. Anti-LAMP-1 mouse monoclonal antibody (H4A3; Developmental Studies Hybridoma Bank), developed under the auspices of the NICHD and maintained by the University of Iowa (Department of Biological Sciences), and was used at a dilution of 1:200. The rabbit polyclonal anti-LAMP-1 antibody 156 was kindly provided by Dr S. Méresse (Centre d'Immunologie de Marseille-Luminy, Marseille, France) and used at a dilution of 1:250. Rabbit polyclonal anti-SseB antibody (Beuzón *et al*., 1999) was used at a dilution of 1:1000. AMCA-, Cy2-, Cy5-, or Rhodamine red X (RRX)-conjugated donkey anti-goat, anti-rabbit or anti-mouse antibodies (Jackson Immunoresearch Laboratories) were used for immunofluorescence at a dilution of 1:400 for Cy5 antibody and 1:200 for the others. Anti-mouse (IgG) and anti-rabbit (IgG) horseradish peroxidase (Amersham Pharmacia Biosciences) were used at a dilution of 1:10 000 for Western blot analysis.

### Cell culture

HeLa (93021013) and RAW 264.7 (91062702) cells were obtained from the European Collection of Cell Cultures, Salisbury, UK. Swiss 3T3 murine fibroblast cells were kindly provided by Dr E. Caron (Imperial College London, UK). Cells were grown in DMEM (Gibco, Carlsbad, CA) supplemented with 10% FCS. Cells were grown at 37°C in 5% CO_2_.

### Bacterial infection of cells and immunofluorescence microscopy and replication assays

HeLa and Swiss 3T3 cells were infected with exponential phase *S*. Typhimurium as described previously (Beuzón *et al*., 2000). Macrophages were infected with opsonized, stationary phase *S.* Typhimurium as described previously (Beuzón *et al*., 2000). To follow a synchronized population of bacteria, host cells were washed after 15 min (HeLa and Swiss 3T3 cells) or 25 min (macrophages) of exposure to *S.* Typhimurium and subsequently incubated in medium containing gentamicin to kill extracellular bacteria. For immunofluorescence, cells were fixed in paraformaldehyde, permeabilized, and incubated with antibodies as described (Beuzón *et al*., 2000). Labelled cells were analysed using a confocal laser scanning microscope (LSM510; Zeiss). For enumeration of intracellular bacteria, macrophages were washed three times with PBS, lysed with 0.1% Triton X-100 for 10 min and dilution series were plated onto LB agar.

### Preparation of protein fractions from bacteria grown *in vitro*

Bacterial cell densities were determined by measurement of the OD_600_ after overnight growth. To ensure that protein from equal numbers of cells was analysed, in all experiments protein samples were adjusted to OD_600_ values such that a volume corresponding to 10 ml of a culture of OD_600_ 0.6 was taken up in 100 μl of protein-denaturing buffer for gel electrophoresis. The secreted and total bacterial cell pellet were prepared as described before (Beuzón *et al*., 1999; Yu *et al*., 2004).

### Protein purification and kinase assay

Plasmids for expression of 6-His fusion proteins (SteC-6His, SteCK256H-6His and C-SteC-6His) were constructed in the pET28b vector using the primers listed in Table S1 and introduced into *E. coli* Rosetta cells (Novagen). Protein expression was induced by using IPTG (0.1 mM) and cells were lysed using French press. Samples were centrifuged at 18 000 *g* for 45 min, and protein samples were passed through a HisTrap HP column (GE Healthcare). Bound proteins were washed and eluted from the column using elution buffer (25 mM Hepes buffer pH 8.0, 500 mM KCl, 5% glycerol) with increasing concentration of imidazole (50–500 mM). Samples were dialysed in 40 mM Tris-HCl, pH 7.4, overnight and used directly for kinase assays. For assays, 10 μg of expressed protein (SteC-6His, SteCK256H-6His or C-SteC-6His) was added to a reagent mixture containing 50 mM Tris-HCl pH 7.5, 10 mM MgCl_2_, 100 mM NaCl, 1 mM Dithiothreitol, 20 μM ATP, 2 μCi [γ^−32^P]ATP (Amersham, 370 MBq ml^−1^, 3000 Ci mmol^−1^), 10 μg MBP (Sigma). The mixture was incubated for 30 min at 30°C and then subjected to SDS-PAGE followed by Coomassie blue staining and autoradiography.

### Transfection of Swiss 3T3 cells

Transfection vectors for expression of c-myc-tagged SteC, SteCK256H and C-SteC were constructed in the pRK5myc vector using the primers listed in Table S1. Myc-tagged active ROCK (ROCK-K) was a gift from Dr E. Caron (Imperial College London, UK). Swiss 3T3 fibroblast cells were seeded onto glass coverslips (12 mm diameter) at a density of 5 × 10^4^ cells ml^−1^, 24 h before transfection. The jetPEI (Autogen Bioclear) protocol for transfection was followed according to manufacturer's recommendations: 3 μg of transfection vector DNA was added to 100 μl of sterile 150 mM NaCl. In a separate tube, 6 μl of jetPEI cations was added to 100 μl of sterile 150 mM NaCl. The jetPEI cations mixture was then added to the transfection vector mix and left for 30 min at room temperature. After incubation, the mixture was added to the cultured Swiss 3T3 cells, centrifuged at 180 *g* for 5 min, and then incubated at 37°C in 5% CO_2_. Cells were transfected for 20 h, after which serum-free medium was added for another 3 h before the cells were fixed in paraformaldehyde, permeabilized and labelled as described above.

### Competitive Index assay

Female BALB/c mice (B and K Universal, Hull, UK) of 18–22 g were used for all infection studies and were challenged either by i.p. or by oral gavage (p.o.) with 0.2 ml of bacteria suspended in physiological saline solution. The bacterial inocula used were 1 × 10^5^ (i.p.) or 1 × 10^8^ (p.o.) cfu of each strain. At least five mice were inoculated per strain mixture for each experiment. Mice were sacrificed 48 h (i.p.) or 4 days (p.o.) after inoculation. Each CI value is the mean of three independent experiments.

## Supplementary material

The following supplementary material is available for this article online:

Table S1: Oligonucleotide primers used in this study.

This material is available as part of the online article from: http://www.blackwell-synergy.com/doi/abs/10.1111/j.1462-5822.2007.01010.x
