# Supplementary material for: SteC is a Salmonella kinase required for SPI-2-dependent F-actin remodelling
Source: Cell Microbiol. 2008 Jan;10(1):20–30. doi: 10.1111/j.1462-5822.2007.01010.x (PMC2253691; doi:10.1111/j.1462-5822.2007.01010.x)
Supplement: Table S1: — Oligonucleotide primers used in this study. [file cmi0010-0020-TableS1.doc]

**Supplementary Materials**

**Table S1.** Oligonucleotide primers used in this study

| Primer | | Sequence |
| --- | --- | --- |
| *steC* mutant | | |
| Δ*STM1698*-F1 | 5’-GAGACATATGCCGTTTACATTTCAGATCGGAAATCATAGTGTGTAGGCTGGAGCTGCTTC-3’ | |
| Δ*STM1698*-R1 | 5’-TAATTCATCCTTTAATACCTTAGCCACAAGAGTCCCTTCCCATATGAATATCCTCCTTAGT-3’ | |
| Complementation and gfp-construct | | |
| *STM1698*-*EcoRI* | | 5’-ATCGAATTCGGACGGATAGCAAGTACGATAG-3’ |
| *STM1698*-*BamHI* | | 5’-ATCGGATCCCGGTAAATCTGTAGCGAATGTGCC-3’ |
| SteC-2HA | | |
| SteC-2HAP1 | | 5’-GAGGAAGGGACTCTTGTGGCTAAGGTATTAAAGGATGAATTAAAAAAATATCCGTATGATGTGCCGGACTATGCGTATCCGTATGATGTTCCTGAT-3’ |
| SteC-2HAP2 | | 5’-TTACATACTACGCCGAACAACGCTAATACGACGGCATGAGCATATGAATATCCTCCTTAGT-3’ |
| Transfection vectors | | |
| pRK5*steC*-P1 | | 5’-ATCGGATCCCCGTTTACATTTCAGATCGGA-3’ |
| pRK5*steC*-P2 | | 5’-ATCGAATTCTCGGTAAATCTGTAGCGAATG-3’ |
| pRK5*steC*-P3 | | 5’-GGCGAATTCTTATTAAGCGCCGCTCTCATAACCAGA-3’ |
| pRK5*steC*-P4 | | 5’-CGCGGATCCATTGATCAGACGGATGCATCACA-3’ |
| Site-directed mutagenesis | | |
| K256H-F | | 5’-GATGACTTTGTTGTTCACATTCCAGTTAAT-3’ |
| K256H-R | | 5’-CTCATTAACTGGAATGTGAACAACAAAGTC-3’ |
| Protein purification SteC-6His | | |
| pET*steC* -P1 | | 5’-ATCGAGACATATGCCGTTTACATTTCAGATCGGA-3’ |
| pET*steC* -P2 | | 5’-GGCGAATTCTTACTATTTTTTTAATTCATCCTTTAATACCTTAGCCACAAGAGTCCCTTCCTCC-3’ |
| pET*steC* -P3 | | 5’-GGCGAATTCTTATTAAGCGCCGCTCTCATAACCAGA-3’ |
| pET*steC* -P4 | | 5’-ATCGGTCCATATGATTGATCAGACGGATGCATC-3’ |
